# Accumulation of foreign polypeptides to rice seed protein body type I using prolamin portion sequences

**DOI:** 10.1007/s00299-016-2097-5

**Published:** 2016-12-27

**Authors:** Ai Sasou, Takanari Shigemitsu, Shigeto Morita, Takehiro Masumura

**Affiliations:** 1 Laboratory of Genetic Engineering, Graduate School of Life and Environmental Sciences, Kyoto Prefectural University, Shimogamo, Kyoto 606-8522 Japan; 2 Biotechnology Research Department, Kyoto Prefectural Agriculture, Forestry and Fisheries Technology Research Center, Kitainayazuma, Seika-cho, Soraku-gun, Kyoto, 619-0244 Japan

**Keywords:** Rice, Protein body type I, Prolamin, Green fluorescent protein

## Abstract

*****Key message***:**

**Rice prolamins are accumulated in endoplasmic reticulum (ER)-derived proteins bodies, although conserved sequences retained in ER are not confirmed. We investigated portion sequences of prolamins that must accumulate in PB-Is.**

**Abstract:**

Rice seed prolamins are accumulated in endoplasmic reticulum (ER)-derived protein body type I (PB-I), but ER retention sequences in rice prolamin polypeptides have not been confirmed. Here we investigated the lengths of the prolamin portion sequences required for accumulation in PB-Is. Of the rice prolamins, we compared 13a and 13b prolamins because the amino acid sequences of these prolamins are quite similar except for the presence or absence of Cys-residues. We also generated and analyzed transgenic rice expressing several prolamin portion sequence-GFP fusion proteins. We observed that in 13a prolamin, when the portion sequences were extended more than the 68th amino acid residue from the initiating methionine, the prolamin portion sequence-GFP fusion proteins were accumulated in PB-Is. In 13b prolamin, when the portion sequences were extended by more than the 82nd amino acid residue from the initiating methionine, the prolamin portion sequence-GFP fusion proteins were accumulated in PB-Is. When those fusion proteins were extracted under non-reduced or reduced conditions, the 13a prolamin portion sequence-GFP fusion proteins in PB-Is were soluble under only the reduced condition. In contrast, 13b prolamin portion sequence-GFP fusion proteins were soluble under both non-reduced and reduced conditions. These results suggest that the accumulation of 13a prolamin in PB-Is is associated with the formation of disulfide bonds and/or hydrophobicity in 13a prolamin polypeptide, whereas the accumulation of 13b prolamin in PB-Is was less involved in the formation of disulfide bonds.

**Electronic supplementary material:**

The online version of this article (doi:10.1007/s00299-016-2097-5) contains supplementary material, which is available to authorized users.

## Introduction

Plant seeds accumulate storage proteins as a nitrogen source for the development of seedlings. Seed proteins are characterized by their solubility: water-soluble albumins, saline-soluble globulins, dilute acid/alkali-soluble glutelins, and alcohol-soluble prolamins (Osborne 1924). In major cereals such as maize, wheat and rice, prolamins are accumulated in endoplasmic reticulum (ER)-derived protein bodies (PBs) as a result of aggregating in the ER, whereas globulins and glutelins are transported to protein storage vacuoles (PSVs).

The major storage proteins in maize and wheat are prolamins (Shewry et al. 1995; Shewry and Halford 2002). For example, maize prolamins (zeins) are synthesized in the ER, leading to the formation of ER-derived PBs (Lending and Larkins 1989). Wheat prolamins (glutenin and gliadin) are synthesized in the ER, and then glutenins aggregate in the ER, whereas gliadins are transported to vacuoles via the Golgi apparatus, resulting in the formation of the protein matrix by the fusion of ER-derived PBs and PSVs (Tosi et al. 2011).

Unlike these cereals, rice seeds possess both ER-derived PBs which store prolamins (referred to protein body type I; PB-I) and PSVs which store globulins and glutelins (referred to protein body type II; PB-II). Although the storage proteins of cereals vary according to each cereal species as described above, cereal prolamins possess two common structural features. The first feature is the presence of conserved domains, and the second is the presence of repeat sequences consisting of specific amino acid residues (Shewry and Halford 2002).

Several studies have shown that conserved domains or repeat sequences in prolamins play a critical role in the aggregation and formation of ER-derived PBs. For example, in maize γ-zein, the repeat sequence of NH_2_-terminal (PPPVHL) and the Cys-rich domain of the COOH-terminal were shown to be important for retaining the ER and forming the ER-derived PBs (Geli et al. 1994). In wheat γ-gliadin, the repeat sequence of NH_2_-terminal (PQQPFPQ) and the sulfur-rich domain of the COOH-terminal domain were suggested to have important roles in the formation of ER-derived PBs (Francin-Allami et al. 2011).

The ability of cereal prolamins to aggregate in the ER has been investigated in not only cereal grains but also other heterologous tissues. When maize γ, β, and δ-zein were expressed in *Nicotiana tabacum* and *Arabidopsis*, these prolamins formed ER-derived PBs in whole plant tissues (Geli et al. 1994; Hoffman et al. 1987; Bagga et al. 1997). In addition, γ-gliadin and HMW-glutenin formed ER-derived PBs in yeast and tobacco BY-2 cells (Rosenberg et al. 1993; Saumonneau et al. 2011).

Rice prolamins also form ER-derived PBs (PB-Is) in both rice endosperm tissues and heterologous tissues such as leaves, roots, rice calli and yeast (Saito et al. 2009; Shigemitsu et al. 2013; Masumura et al. 2015). However, there is no repeat sequence in rice prolamins that seems to be important in the formation of ER-derived PBs in other cereals. Kawagoe et al. (2005) demonstrated that the formation of disulfide bonds played a critical role in the aggregation of prolamins in PB-Is, but rice seed 13b prolamins lack the Cys-residue that is essential to form disulfide bonds (Saito et al. 2012). Shigemitsu et al. (2013) showed that 13b prolamin-GFP fusion proteins aggregated into ER-derived PBs, the same as other prolamin-GFP fusion proteins do in transgenic rice calli. Thus, the unique repeat sequences and formations of disulfide bonds by Cys-residues are not sufficient to explain the mechanism underlying the accumulation of the rice prolamins in PB-Is.

Although some questions remain to be answered, it appears that the ability of prolamins to aggregate in the ER can be adapted as the ER accumulation sequences for the production of recombinant proteins in plant tissues. Gutiérrez et al. (2013) showed that when recombinant proteins were expressed as the fusion proteins with elastin-like polypeptide and hydrophobin in plant tissues, the quantities of the proteins were likely to increase and many ER-derived PBs were induced. It is thus an efficient strategy to express the recombinant proteins in the ER for high production in pant tissues.

**Fig. 1:**
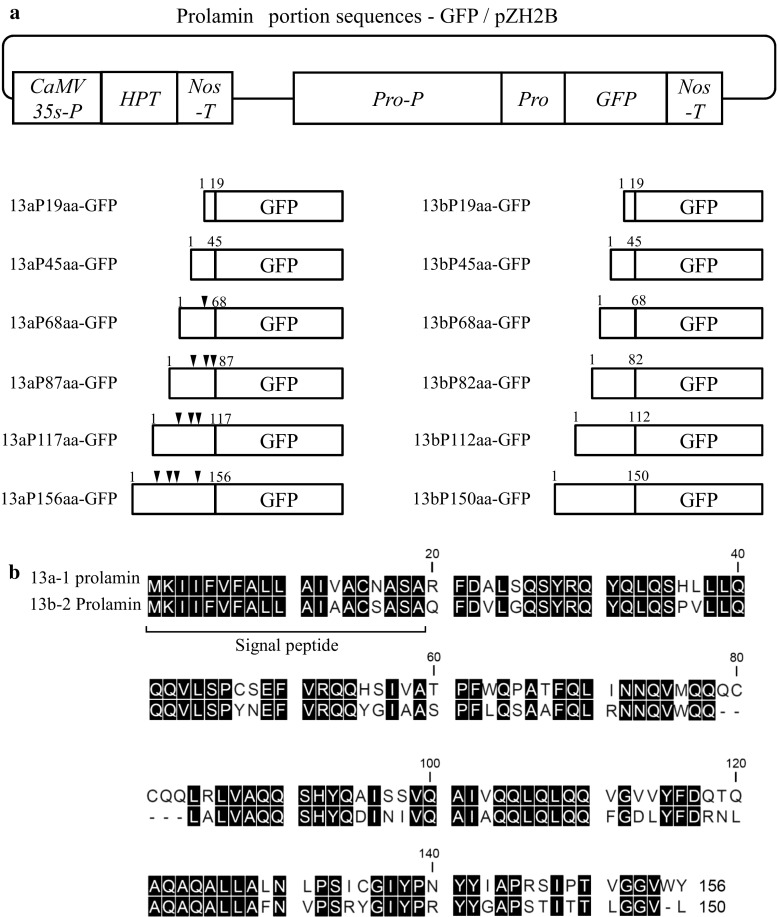
**a** The constructs expressing prolamin portion sequence-GFP fusion proteins in transgenic rice seeds. *CaMV35S-P* cauliflower mosaic virus 35S promoter, *HPT* hygromycin phosphotransferase, *Nos-T* nopaline synthase terminator, *Pro-P* prolamin promoter, *Pro* prolamin, *GFP* green fluorescent protein; arrowheads indicate the position of cysteine residues. **b** Comparison of the amino acid sequences of 13a-1 and 13b-2 prolamins. *Upper* sequence: 13a-1 prolamins (λRM1, Os07g0206500). *Lower* sequences: 13b-2 prolamin (λRM4, Os05g0329300). *White letters* on a *black* background indicate the same amino acid between 13a-1 prolamin and 13b-2 prolamin. Portion sequences from initiating methionine to 19th amino acid in 13a/13b prolamin polypeptides are signal peptides

In our previous study, we showed that when each prolamin-GFP fusion protein was expressed under the control of each native prolamin promoter, the GFP accumulation was in the same region as the endogenous prolamin location (Sasou et al. 2016). We also proposed the utilization of PB-Is as the carrier of oral vaccines in light of the indigestibility of PB-Is in the human alimentary tract. In the present study, we investigated the length of portion sequences of rice prolamin polypeptides to accumulate in PB-Is by comparing 13a prolamin with 13b prolamin. The amino acid sequences of 13a prolamin and 13b prolamin are quite similar except for the presence or absence of Cys-residues (Fig. 1b). Regarding the presence or absence of Cys-residues and the length of prolamin portion sequences, we, therefore, examined whether 13a/13b prolamin portion sequence-GFP fusion proteins are accumulated in PB-Is of transgenic rice seeds in the present study. We speculated that the essential sequences of prolamin polypeptides to accumulate in PB-Is are used as the ER accumulation sequences when the foreign proteins are produced in transgenic rice seeds.

## Materials and methods

### Constructs

The constructs expressing each prolamin portion sequence-GFP fusion protein were generated based on pZH2B vector (Kuroda et al. 2010). The DNA fragment of GFP-NOS was digested from the GFP-NOS/pUC198AM plasmid (Saito et al. 2012) with the use of *Bam*HI and *Eco*RI and was inserted into pZH2B (GFP/pZH2B). The promoter and portion sequences of 13a-1 prolamin and 13b-2 prolamin were amplified from 13a-1P-GFP/pZH2B and 13b-2P-GFP/pZH2B plasmid as the template (Sasou et al. 2016) using the primer set shown in Table 1.

**Table 1:** Primer sequences used to generate the constructs expressing the prolamin portion sequence-GFP fusion proteins

Primer name	Upstream and downstream primer sequences
Forward primer for 13a prolamin	5′-ATATCTAGAGTTGAAGCATAGTAGAATCC-3′
Reverse primer for 13a prolamin portion sequences	
1–19 aa	5′-ATAGGATCCTGCAGAAGCGTTGCATGCAA-3′
1–45 aa	5′-ATAGGATCCGCTGAGCACTTGTTGCTGTA-3′
1–68 aa	5′-ATAGGATCCAAACGTAGCTGGTTGCCAGA-5′
1–78 aa	5′-ATAGGATCCTACCAGCCTGAGCTGTTGGC-3′
1–117 aa	5′-ATAGGATCCATCAAAGTAGACAACAACGA-3′
1–156 aa (full length)	5′-TAGGATCCGTACCAGACACCACCAACGG-3′
Forward primer for 13b prolamin	5′-GCGTCTAGAGCATTATACAGCAAAATAGA-3′
Reverse primer for 13b prolamin portion sequences	
1–19 aa	5′-ATAGGATCCCGCAGAGGCGCTGCATGCA-3′
1–45 aa	5′-ATAGGATCCGCTAAGCACCTGTTGCTGTA-3′
1–68 aa	5′-ATAGGATCCAAACGCAGCTGATTACAAGA-3′
1–72 aa	5′-ATAGGATCCCACCAGCGCGAGCTGTTGC-3′
1–112 aa	5′-ATAGGATCCATCAAAGTAGAGATCACCAA-3′
1–150 aa (full length)	5′-ATAGGATCCGTACCTAGGGTAGATACCAT-3′

These PCR fragments were digested with restriction endonuclease and then inserted upstream of the GFP sequences of the GFP/pZH2B vector. We named the resulting binary vectors 13aP19aa-GFP (signal peptide of 13a prolamin-GFP), 13aP45aa-GFP, 13aP68aa-GFP, 13aP87aa-GFP, 13aP117aa-GFP, 13aP156aa-GFP (full length of 13a prolamin-GFP), 13bP19aa-GFP (signal peptide of 13b prolamin-GFP), 13bP45aa-GFP, 13bP68aa-GFP, 13bP82aa-GFP, 13bP112aa-GFP, and 13bP150aa-GFP (full length of 13b prolamin-GFP), respectively (Fig. 1a).

We introduced these binary vectors into rice calli using the *Agrobacterium*-mediated method as described (Hiei et al. 1994). The rice calli containing the transgene were selected by hygromycin B (Nacalai Tesque, Kyoto, Japan) and transferred to redifferentiation medium. The shoots from selected calli were then transferred to 1/5000 a Wagner pots containing soil. We then analyzed T_2_ transgenic rice seeds of three independent lines in each construct.

### Plant material and growth conditions

The transgenic rice plants were grown in Wagner pots with soil in a naturally illuminated temperature-controlled (28 °C) greenhouse in the Biotechnology Research Department, Kyoto Prefectural Agriculture, Forestry, and Fisheries Technology Research Center, Japan.

### Protein extraction and immunoblotting

For the extraction of total protein, the flour of mature seeds was homogenized in sodium dodecyl sulfate (SDS) sample buffer [62.5 mM Tris–HCl (pH 6.8), 4 M urea, 2% (w/v) SDS] supplemented with or without 0.1 M dithiothreitol (DTT) for 1 h. The homogenates were centrifuged at 15,000*g* for 5 min to obtain the protein extracts as supernatant solutions. The extracts were then heated at 100 °C for 5 min. The separated polypeptides after SDS-PAGE were electrotransferred to an Immun-Blot PVDF Membrane (Bio-Rad, Hercules, CA, USA), revealed using anti-GFP antibody (dilution 1:5000; Medical & Biological Laboratories, Nagoya, Japan), and reacted with the alkaline phosphatase (AP)-conjugated goat anti-rabbit IgG secondary antibody (1:20,000; Promega, Madison, WI, USA). AP-labeled bands were detected with 5-bromo-4-chloro-3-indoyl phosphate and nitroblue tetrazolium using BCIP/NBT Color Development Substrate (Promega) according to the manufacturer’s instructions.

### Protein extraction using sample buffer without SDS

For the extraction using sample buffer without SDS, the flour of mature seeds was homogenized in sample buffer (-SDS) [62.5 mM Tris–HCl (pH 6.8), 4 M urea] supplemented with 0.1 M dithiothreitol (DTT) for 1 h. The homogenates were centrifuged at 15,000*g* for 5 min to obtain the protein extracts as supernatant solutions. For the separation by SDS-PAGE, the protein extracts were mixed with equal quantity of 2× sample buffer [125 mM Tris–HCl (pH 6.8), 8 M urea, 0.2 M DTT]. After that, SDS-PAGE and immunoblotting analysis were performed by the procedure described in section of protein extraction and immunoblotting.

### Fluorescence microscopic analysis

The thin sections of mature seeds were prepared in accordance with the frozen film method described by Saito et al. (2008). The prepared thin sections (2 μm) were stained for 10 min with 10 nM rhodamine B (Wako Pure Chemical Industries, Osaka, Japan), which is a pigment that stains the peripheral region of PB-I (Choi et al. 2000). Stained sections were observed with a fluorescence microscope (BX51; Olympus, Tokyo), and the images were analyzed with an Aquacosmos system (Hamamatsu Photonics, Hamamatsu, Japan).

### Comparison of hydrophilicity between 13a prolamin and 13b prolamin

We produced a hydropathy plot with the gene information processing software GENETYX ver.13 by the method developed by Hopp and Woods (1981). The serial average of the hydrophilicity value assigned to each amino acid with pentapeptide was calculated and is shown in the graph (Fig. 6). The area filled red in the graphs indicate the hydrophobic area.

## Results

### The accumulation of prolamin portion sequence-GFP fusion proteins

**Fig. 2:**
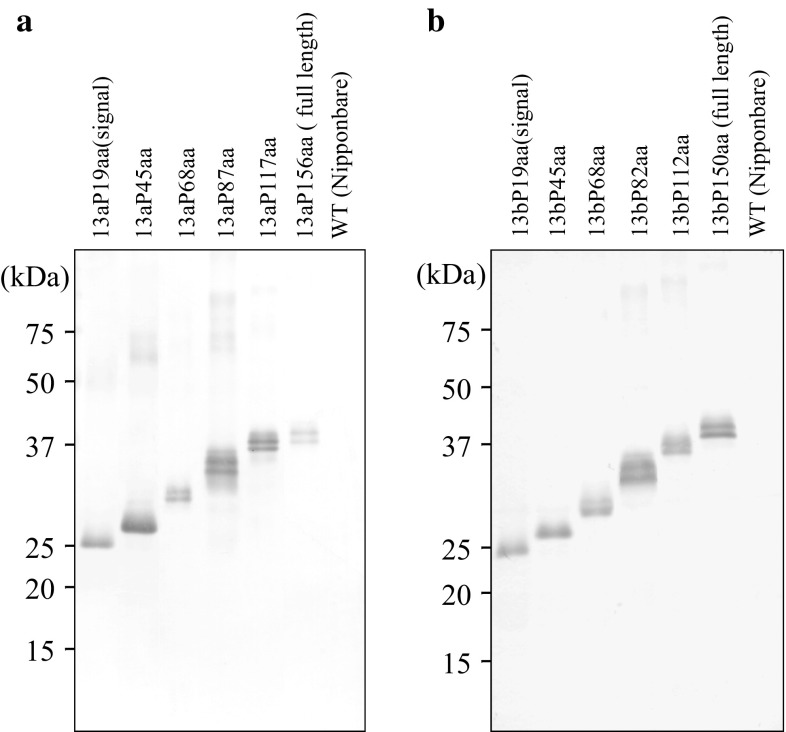
Accumulation of each prolamin portion sequence-GFP fusion protein in the transgenic rice seeds. Each fusion proteins were extracted under reducing condition using the sample buffer [62.5 mM Tris–HCl (pH 6.8), 4 M urea, 2% (w/v) SDS, 0.1 M dithiothreitol (DTT)]. The immunoblot analysis was performed using anti-GFP antibody in transgenic rice seeds expressing 13a portion sequence-GFP fusion proteins (**a**) and 13b portion sequence-GFP fusion proteins (**b**)

The accumulation of each prolamin portion sequence-GFP fusion protein in transgenic rice was confirmed by the immunoblot analysis with anti-GFP antibody. This analysis revealed the bands of each fusion protein in the transgenic rice seeds: the molecular sizes of 13aP19aa-GFP and 13bP19aa-GFP were 27 kDa; those of 13aP45aa-GFP and 13bP45aa-GFP were 29 kDa; those of 13aP68aa-GFP and 13bP68aa-GFP were 32 kDa; those of 13aP87aa-GFP and 13bP82aa-GFP were 34 kDa; those of 13aP117aa-GFP and 13bP112aa-GFP were 37 kDa, and those of 13aP156aa-GFP and 13bP150aa-GFP were 40 kDa (Fig. 2).

In the 13aP87aa-GFP, 13aP117aa-GFP, 13aP156aa-GFP, 13bP82aa-GFP, 13bP112aa-GFP and 13bP150aa-GFP transgenic rice seeds, the fusion proteins were detected as doublet bands. The bands of predictable molecular sizes were upper bands, respectively. These lower bands imply the processed form of each fusion protein.

### The accumulation of prolamin portion sequence-GFP fusion proteins in the PB-Is of each transgenic rice seed

**Fig. 3:**
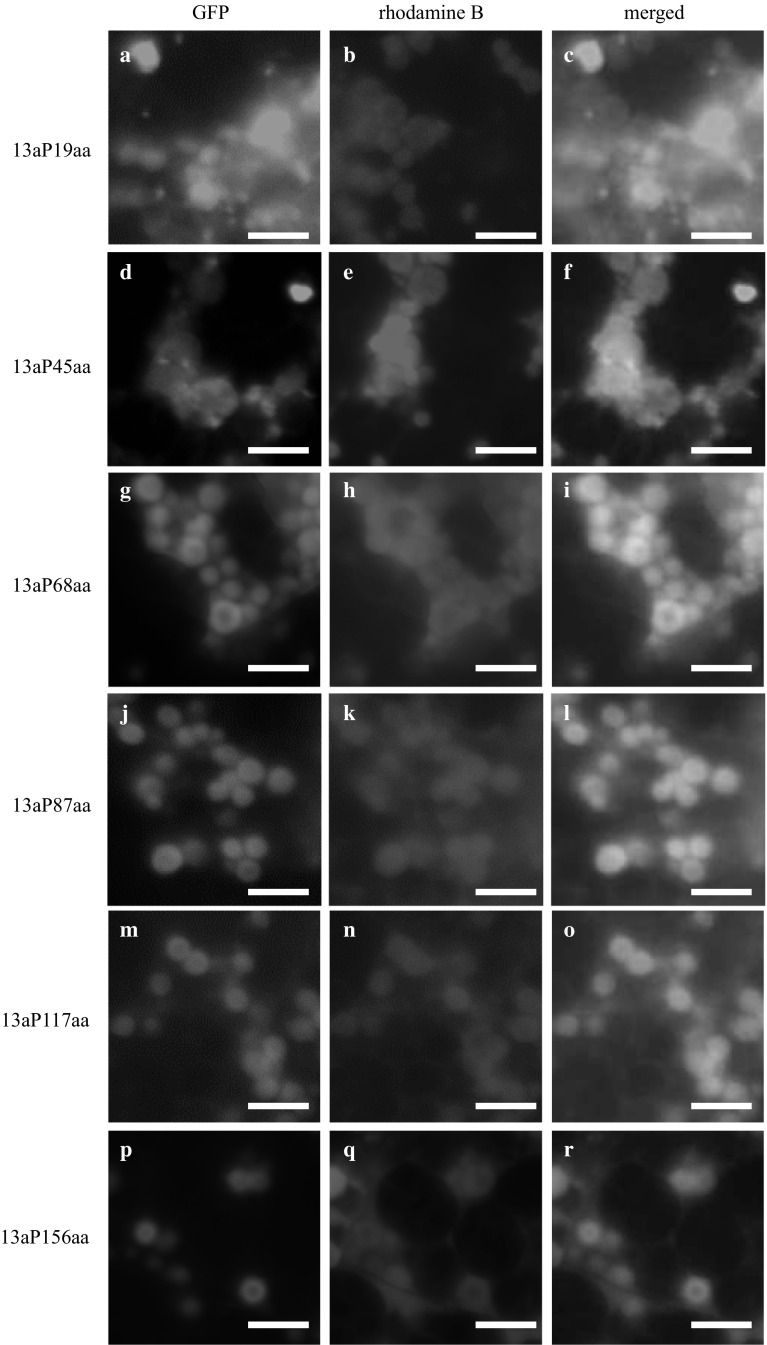
The accumulation of the portion sequences of 13a prolamin-GFP fusion protein. The fluorescence images of mature transgenic rice seeds expressing the 19th amino acid portion sequence-GFP fusion protein (**a**–**c**), the 45th amino acid portion sequence-GFP fusion protein (**d**–**f**), the 68th amino acid portion sequence-GFP fusion protein (**g**–**i**), the 87th amino acid portion sequence-GFP fusion protein (**j**–**l**), the 117th amino acid portion sequence-GFP fusion proteins (**m**–**o**), and the 156th amino acid sequence (full length)-GFP fusion protein (**p**–**r**) were captured by fluorescence microscopy. Green images: GFP fluorescent signals (**a**, **d**, **g**, **j**, **m**, **p**). *Magenta* images: the fluorescence of rhodamine B, i.e., the pigment staining the peripheral region of PB-Is (**b**, **e**, **h**, **k**, **n**, **q**). The merged images indicate **c**, **f**, **i**, **l**, **o** and **r**. *Bars* 5 μm

The fluorescence microscopic analysis revealed the accumulation of the 13a prolamin portion sequence-GFP fusion proteins (Fig. 3). The accumulation of fusion proteins was inside the ER, but not in PB-Is of the 13aP19aa-GFP or 13aP45aa-GFP lines (Suppl. Figure 1, Fig. 3a–c, d–f, respectively). On the other hand, the accumulation of fusion proteins were in the middle layer of the PB-Is in the 13aP68aa-GFP line (Fig. 3g–i). GFP with the extended portion sequences more than the 68th amino acid of 13a prolamin (87, 117 and 156 aa) was also accumulated in the middle layer of the PB-Is (Fig. 3j–l, m–o, p–r, respectively).

**Fig. 4:**
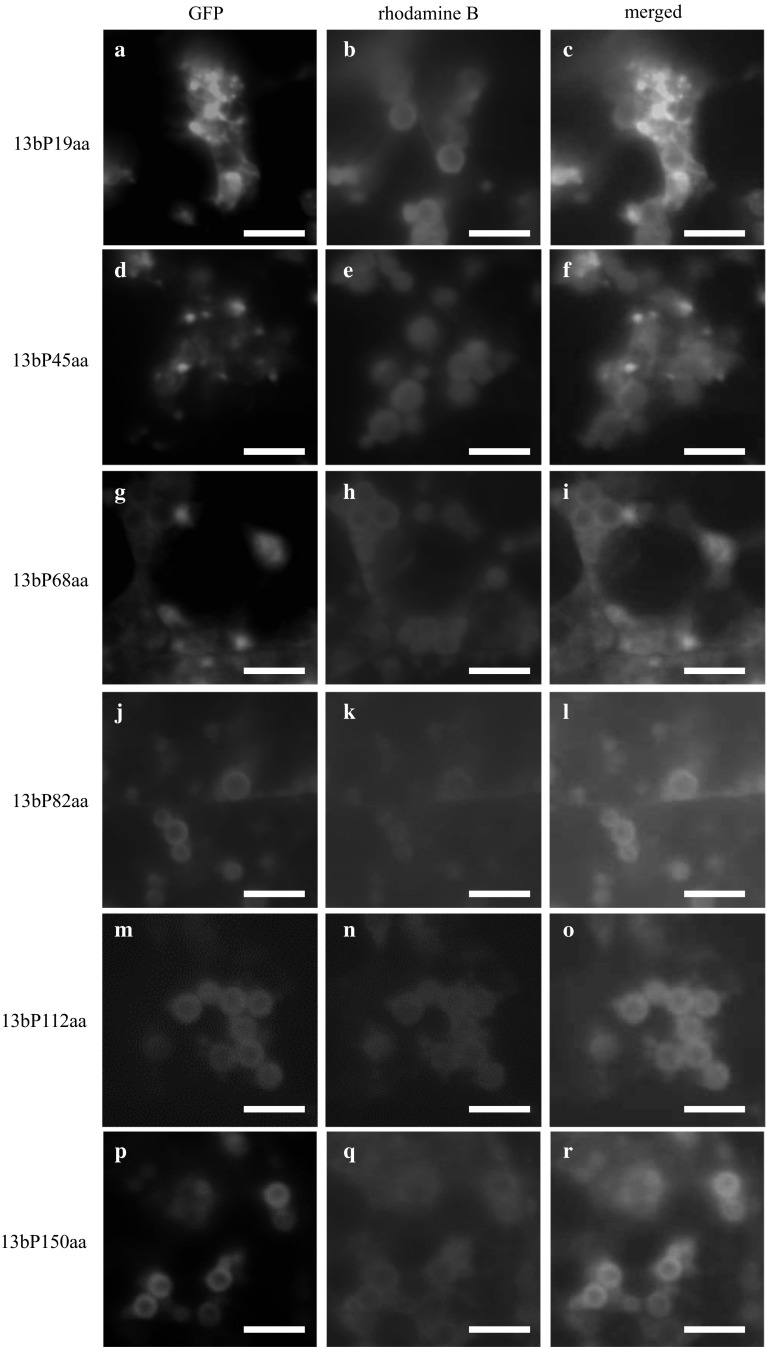
The accumulation of the portion sequences of 13b prolamin-GFP fusion protein. The fluorescence images of mature transgenic rice seeds expressing the 19th amino acid portion sequence-GFP fusion protein (**a**–**c**), the 45th amino acid portion sequence-GFP fusion protein (**d**–**f**), the 68th amino acid portion sequence-GFP fusion protein (**g**–**i**), the 82nd amino acid portion sequence-GFP fusion protein (**j**–**l**), the 112nd amino acid portion sequence-GFP fusion protein (**m**–**o**), and the 150th amino acid sequence (full length)-GFP fusion protein (**p**–**r**) were captured by fluorescence microscopy. Green images: GFP fluorescent signals (**a**, **d**, **g**, **j**, **m**, **p**). *Magenta* images: the fluorescence of rhodamine B, i.e., the pigment staining the peripheral region of PB-Is (**b**, **e**, **h**, **k**, **n**, **q**). The merged images indicate **c**, **f**, **i**, **l**, **o**, and **r**. *Bars* 5 μm

In the same manner as 13a prolamin, the accumulation of 13b prolamin portion sequence-GFP fusion proteins was demonstrated by the fluorescence microscopic analysis (Fig. 4). The accumulation of fusion proteins was inside the ER, but not in the PB-Is of the 13bP19aa-GFP, 13bP45aa-GFP, or 13bP68aa-GFP lines (Suppl. Figure 2, Fig. 4a–c, d–f, g–i, respectively). The accumulation of fusion proteins was in the outermost layer of the PB-Is in 13bP82aa-GFP (Fig. 4j–l). GFP with the extended portion sequences more than the 82nd amino acid of the 13b prolamin (112 and 150 aa) was also accumulated in the outermost layer of the PB-Is (Fig. 4m–o, p–r, respectively).

### Protein extraction under the reduced or non-reduced conditions in transgenic rice seeds

**Fig. 5:**
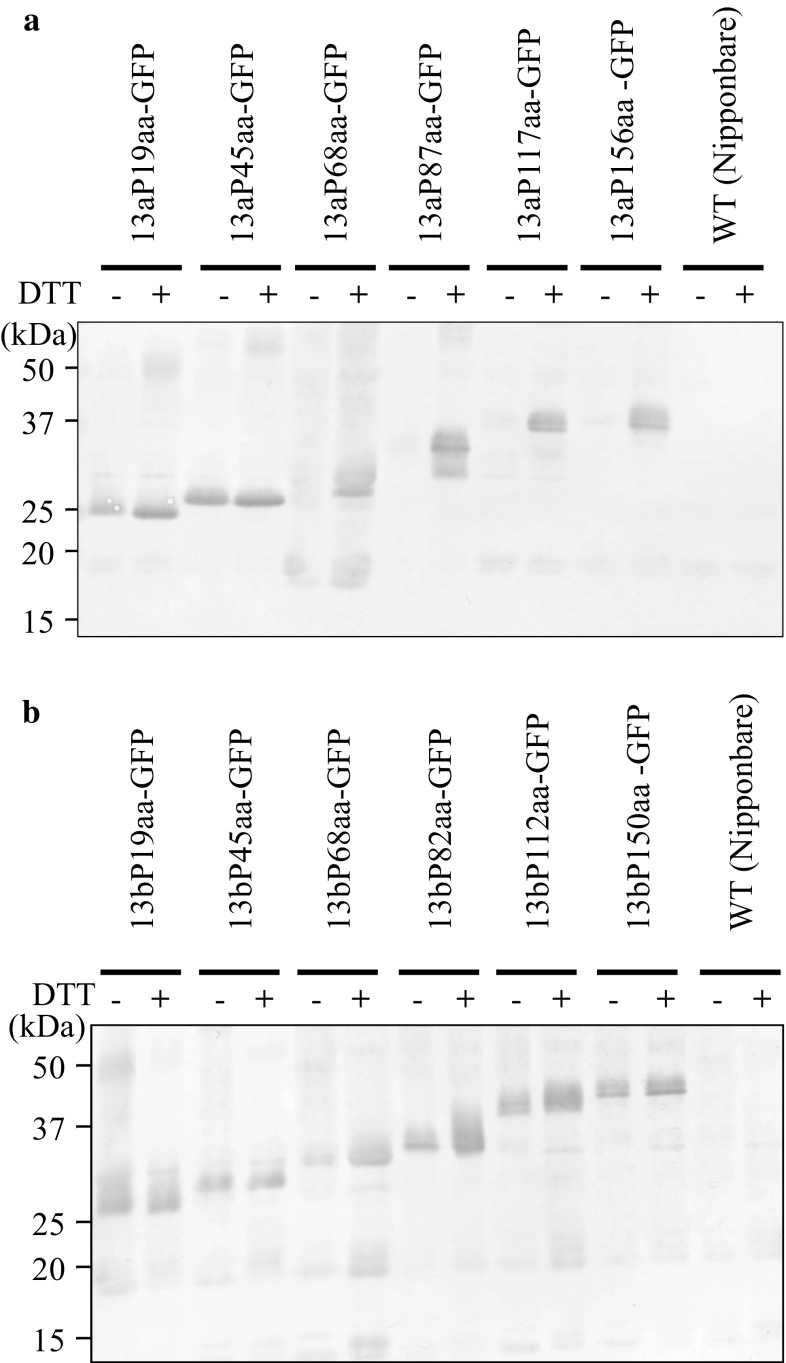
The extraction and detection of each fusion protein under the non-reduced or reduced conditions. The extractions under non-reduced conditions or reduced conditions were performed with sample buffer with or without DTT. The* symbol* “−” indicates the extraction using sample buffer without DTT, and “+” indicates the extraction with DTT. The detection of each 13a prolamin portion sequence-GFP fusion protein (**a**) or 13b portion sequence-GFP fusion protein (**b**) in transgenic rice seeds was confirmed by immunoblotting using anti-GFP antibody

The solubility of each prolamin portion sequence-GFP fusion protein was investigated by the extraction of sample buffer with or without DTT. In 13aP19aa-GFP and 13aP45aa-GFP, the fusion proteins were extracted under the reduced and non-reduced conditions. In contrast, in 13aP68aa-GFP, 13aP87aa-GFP, 13aP117aa-GFP, and 13aP156aa-GFP, the fusion proteins were only extracted under the reduced condition (Fig. 5a). All of the 13b prolamin portion sequence-GFP fusion proteins were extracted under both the reduced and non-reduced conditions (Fig. 5b).

### Protein extraction using sample buffer without SDS

The dissolution of prolamin portion sequence-GFP fusion proteins in sample buffer without SDS was confirmed using sample buffer without SDS and the immunoblot analysis with anti-GFP antibody. SDS helps the dissolution of the hydrophobic proteins into the buffer, so that hydrophobic proteins cannot be extracted without SDS. In 13aP19aa-GFP, 13aP45aa-GFP, 13aP68aa-GFP, and 13aP87aa-GFP, the fusion proteins were extracted using sample buffer without SDS. While in 13aP117aa-GFP and 13aP156aa (full length)-GFP, the fusion proteins could not be extracted under the condition without SDS (Suppl. Figure 3a). In 13bP19aa-GFP, 13bP45aa-GFP, 13bP68aa-GFP, and 13bP82aa-GFP, the fusion proteins were extracted using sample buffer without SDS. On the other hand, in 13bP112aa-GFP and 13bP150aa (full length)-GFP, the fusion proteins could not be extracted under the condition without SDS (Suppl. Figure 3b).

### Comparison of hydrophilicity in polypeptides between 13a prolamin and 13b prolamin

**Fig. 6:**
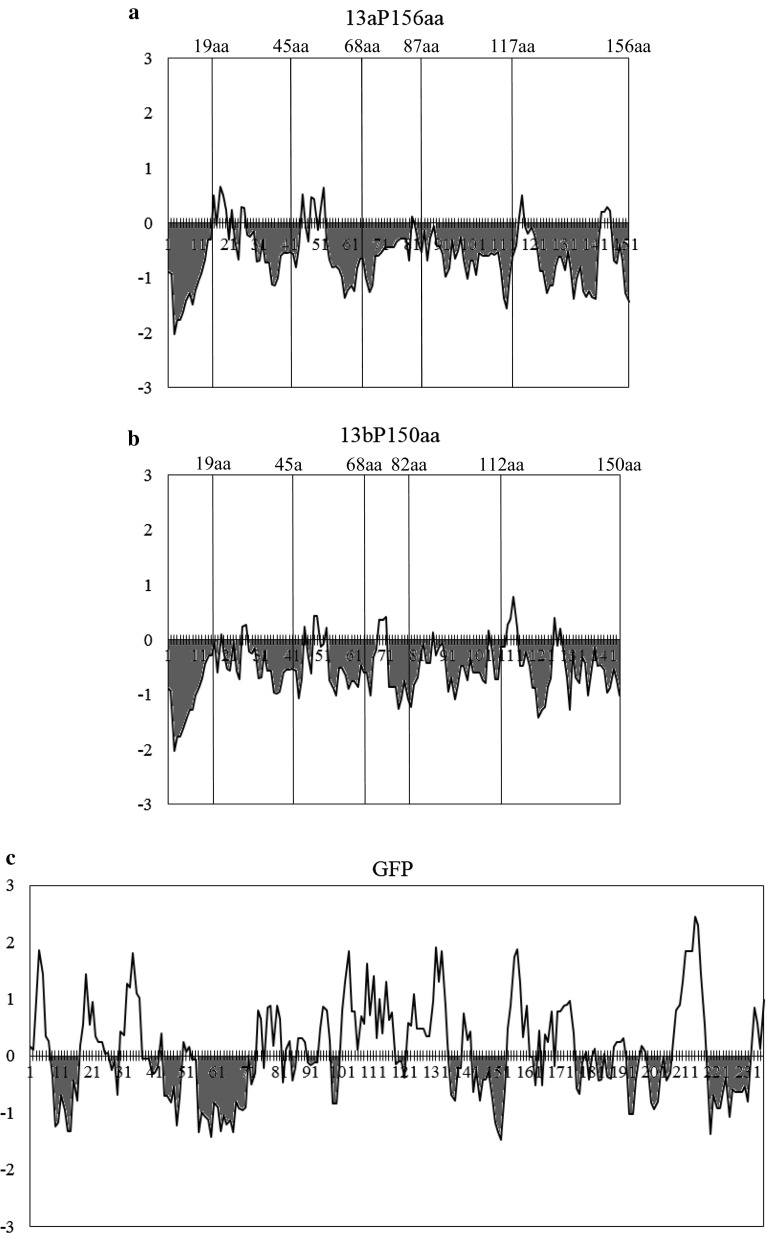
Hopp and Woods hydropathy plot analysis. The horizontal axis indicates the sequence position in 13a prolamin (**a**), 13b prolamin (**b**) and GFP (**c**) polypeptides, and the vertical axis indicates the hydrophilicity value. When the average of the hydrophilicity value in the pentapeptide is positive, this region is hydrophilic. When the average of the hydrophilicity value in the pentapeptide is negative, this region is hydrophobic (filled by *red*). *Vertical lines* in panels **a** and **b** indicate the positions at the end of each portion sequence (color figure online)

The hydropathy plot of 13a/13b prolamins is provided in Fig. 6. The hydropathy plots showed that 13a and 13b prolamins are entirely hydrophobic, and the patterns of the graphs were similar (Fig. 6a, b); the plots also showed that GFP is a hydrophilic protein (Fig. 6c). These results indicate that the portion sequences of 13a and 13b prolamins gradually became hydrophobic when the portion sequences became longer from the initiating methionine.

## Discussion

We investigated portion sequences in prolamin polypeptides that were essential to accumulation in PB-Is. Both 13a and 13b prolamins possess no unique repeat sequences that are recognized in other cereal prolamins. In addition, 13b prolamins possess no Cys-residues to form the intramolecular or intermolecular disulfide bonds. We, therefore, examined whether 13a/13b prolamin portion sequence-GFP fusion proteins were accumulated in the PB-Is of transgenic rice seeds by performing a comparison of the presence or absence of Cys-residues and the length of 13a/13b prolamin portion sequences. The results showed that the 13a prolamin portion sequence-GFP fusion proteins (13aP68aa-GFP) and the 13b prolamin portion sequence-GFP fusion proteins (13bP82aa-GFP) became accumulated in the PB-Is (Figs. 3, 4). In 13aP68aa-GFP, one Cys-residue is present in the 13a prolamin portion sequence. There is no Cys-residue in the 13b portion sequence of the mature protein in 13bP82aa-GFP. Thus, in the case of the 13a prolamin 68th portion sequence, the accumulation of GFP fusion proteins in PB-Is could be affected by the formation of disulfide bonds. In the case of the 13b prolamin 82nd portion sequence, we suspect that the accumulation of GFP fusion proteins in PB-Is is affected by other factors such as hydrophobicity in the 13b prolamin portion sequence. This result suggests that rice prolamins can accumulate in PB-Is without a particular signal for retention in the ER. In addition, Cys-residues are not always required to accumulate in PB-Is as the case of 13b prolamins.

In a study by Sasou et al. (2016), the full-length 13a prolamin-GFP fusion proteins (13a-1P-GFP) were localized in the middle layer of the PB-Is, and the full-length 13b prolamin-GFP fusion proteins (13b-2P-GFP) were localized in the outermost layer of the PB-Is. In the present study, the 13a portion sequences at more than the 68th amino acid fused with GFP seemed to be localized in the middle layer and the 13b portion sequences more than the 82nd amino acid fused with GFP seemed to be localized in the outermost layer of PB-Is. The layer structure of PB-Is is thought to be involved in the expression order of each prolamin during the development of rice seeds by the function of its promoter (Saito et al. 2012). It, therefore, appears that prolamin portion sequences are less likely to alter the layer structure.

The experiment of the protein extraction from each transgenic rice seed under the reduced or non-reduced conditions showed that the accumulation of the 13a portion sequence more than the 68th amino acid in the PB-Is was associated with the formation of disulfide bonds (Fig. 5a). In contrast, the accumulation of the 13b portion sequence more than the 82nd amino acid was affected by a factor other than the formation of disulfide bonds (Fig. 5b). In the 13a prolamin 68th amino acid portion sequence, there is one Cys-residue in the mature protein, and we, therefore, speculated that the accumulation of 13a prolamin in PB-Is is associated with the formation of disulfide bonds and/or hydrophobicity in 13a prolamin polypeptides. Normally, site-directed mutagenesis of Cys-residues experiments may be required to demonstrate which species of endogenous prolamins form disulfide bonds with 13a prolamin portion sequence-GFP fusion protein. However, 13a and 13b prolamins were compared to confirm the effect on accumulation each prolamin in PB-Is with or without Cys-residues in present study.

13b prolamin portion sequence-GFP fusion proteins of 13bP82aa-GFP or more extended portion sequences of 13b portion sequences became accumulated in the outermost layer of the PB-Is without a Cys-residue. The results of our experiment regarding the protein extraction from each transgenic rice seed using sample buffer without SDS suggested that the hydrophobicity of each prolamin portion sequence-GFP fusion protein became stronger when the lengths of the prolamin portion sequences were extended from the initiating methionine (Suppl. Figure 3). 13b Prolamin (full length)-GFP fusion proteins were observed to have the ability to form small PBs derived from the ER in transgenic rice calli despite a lack of Cys-residues in 13b prolamins (Shigemitsu et al. 2013). Our findings from these extraction experiments suggest that the accumulation of 13b prolamin in PB-Is is associated with the hydrophobicity in 13b prolamin polypeptides without formation of disulfide bonds. In the 13aP68aa-GFP, 13aP87aa-GFP, 13bP82aa-GFP, and 13bP112aa-GFP binary vectors, part of the portion sequence-GFP fusion proteins could be extracted by sample buffer without SDS despite the accumulation of fusion protein in the PB-Is. We suspect that the reason for this is that the hydrophilicity of GFP affected the hydrophilic/hydrophobic characteristics in the fusion proteins.

In the hydropathy plot analysis, both 13a and 13b prolamin portion sequences showed high hydrophobicity in each polypeptide (Fig. 6a, b, the area filled by red). These hydropathy plots revealed that the hydrophobicity between 13a and 13b prolamins showed almost the same pattern. The hydropathy plot analysis also indicated that the hydrophobicity of the 68th amino acid sequence between 13a prolamin and 13b prolamin showed the same pattern. However, the 68th portion sequence of 13a prolamin possesses a Cys-residue, and the 13aP68aa-GFP fusion protein began to accumulate in PB-Is by the formation of a disulfide bond (Fig. 5a). On the other hand, the 82nd amino acid or longer sequences of 13b prolamins became hydrophobic enough to accumulate in ER-derived PB-Is (Fig. 4j–r). Instead of the disulfide bonds, the hydrophobic degree in 13b prolamin polypeptides is thought to be associated with the accumulation in PB-Is.

Masumura et al. (2015) showed the accumulation of 13a portion sequences (20–68 aa, 69–117 aa, 111–156 aa)-GFP fusion proteins in the yeast. In the yeast, the N-terminal sequence (20–68 aa) of 13a prolamin polypeptide fused with GFP accumulated in the vacuole, while the middle sequence (69–117 aa) and C-terminal sequence (111–156 aa) of 13a prolamin polypeptides fused with GFP accumulated in the ER without forming disulfide bonds. In 13a prolamin, the hydrophobic region of middle sequence (69–117 aa) and C-terminal sequence (111–156 aa) are larger than that of N-terminal sequence (20–68 aa) (Fig. 6a). These findings indicate that the middle and C-terminal portion sequences may function to accumulate in the ER due to the characteristics of hydrophobicity in prolamin polypeptides. If we also perform the N-terminal deletion, shorter sequences to accumulate in the ER could be found in the middle or C-terminal region of prolamins.

When the foreign proteins were synthesized and accumulated in a specific layer of PB-Is in rice seeds, the portion sequences of 13a and 13b prolamin seem to be used for the ER accumulation sequences, the same as the full-length 13a and 13b prolamins. Shigemitsu et al. (2013) showed that GFP fused with full-length 13a or 13b prolamin was accumulated in the ER-derived PBs of transgenic rice calli. The portion sequences of 13a or 13b prolamins can, therefore, be used as the ER accumulation sequences not only in transgenic rice seeds, but also in other heterologous expression systems. The utilization of prolamin portion sequences is expected to provide a technique to protect vaccine antigens expressed in transgenic plants from gastric acid and protease enzymes when such vaccine antigens are administered orally.

### **Author contribution statement**

TM designed this research. TS contributed to the generation of transgenic rice. SM conducted and discussed the result of the immunoblot and fluorescent microscopic analysis. AS performed the experiments and wrote this manuscript. All authors read and approved the manuscript.

## Electronic supplementary material

Below is the link to the electronic supplementary material.

Supplementary material 1 (PDF 170 kb)

## Abbreviations

Cys: Cysteine
ER: Endoplasmic reticulum
GFP: Green fluorescent protein
PB-I: Protein body type I
PB-II: Protein body type II
PCR: Polymerase chain reaction
PSV: Protein storage vacuole
SDS-PAGE: Sodium dodecyl sulfate–polyacrylamide gel electrophoresis
WT: Wild type
