# Supplementary material for: Accumulation of foreign polypeptides to rice seed protein body type I using prolamin portion sequences
Source: Plant Cell Rep. 2016 Dec 27;36(3):481–91. doi: 10.1007/s00299-016-2097-5 (PMC5316557; doi:10.1007/s00299-016-2097-5)
Supplement: Supplementary file 1 — Supplementary material 1 (PDF 170 kb) [file 299_2016_2097_MOESM1_ESM.pdf]

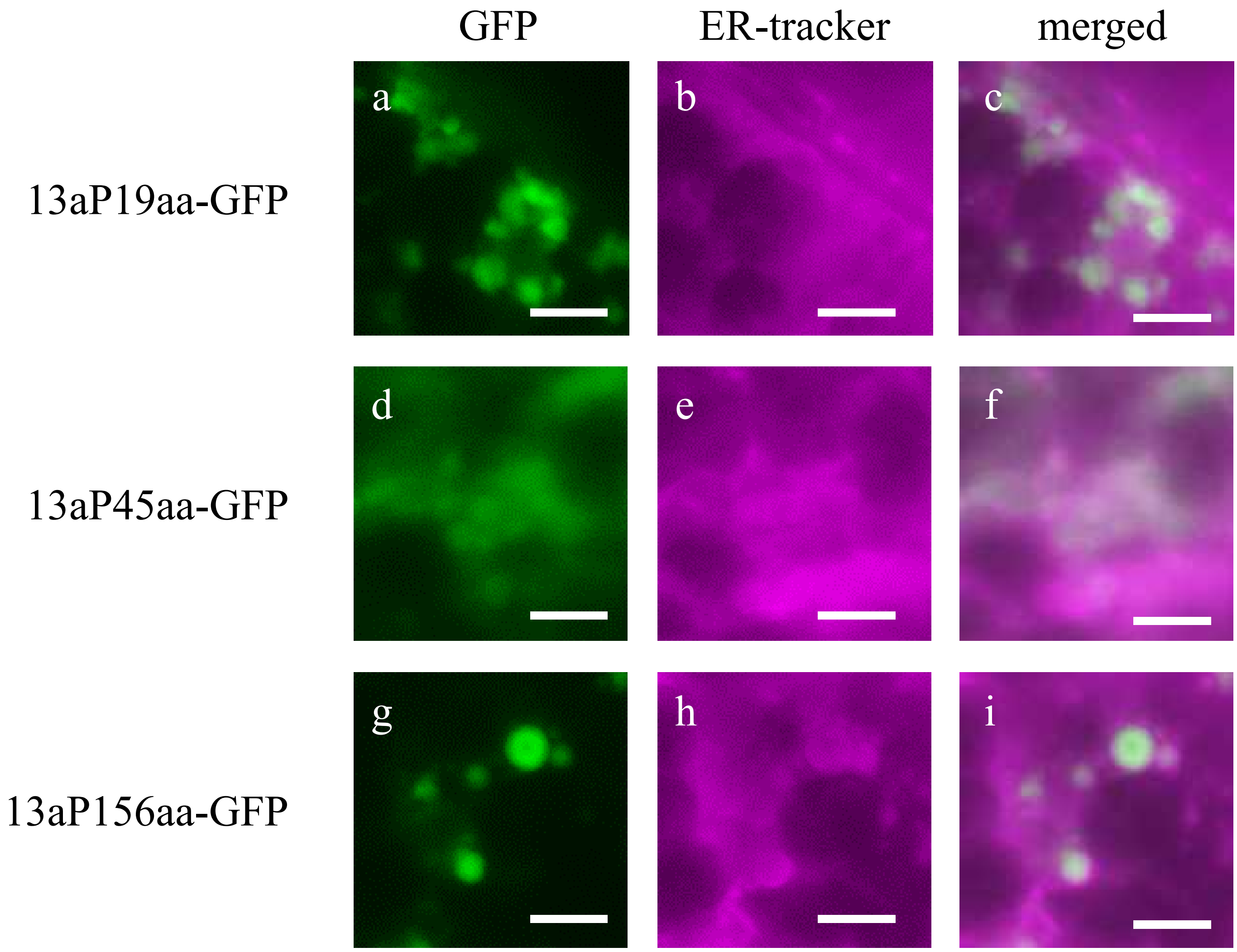

Supplementary Fig. 1. The accumulations of 13a prolamin portion sequence-GFP fusion proteins in 13aP19aa-GFP and 13aP45aa-GFP were in the ER. We stained frozen thin sections with 10 nM ER-Tracker™ Red (Invitrogen, Carlsbad, CA) for 20 min. The fluorescence images of mature transgenic rice seeds expressing the 19th amino acid (aa) portion sequence-GFP fusion protein (**a**–**c**), the 45th aa portion sequence-GFP fusion protein (**d**–**f**), and the 156th aa sequence (full length)-GFP fusion protein (**g**–**i**) were captured by fluorescence microscopy. Green images: GFP fluorescent signals (**a**, **d**, **g**). Magenta images: the fluorescence of ER-Tracker™ Red (**b**, **e**, **h**). The merged images are **c**, **f**, and **i**. The signals of GFP are surrounded by ER-Tracker. Bars = 5 μm.

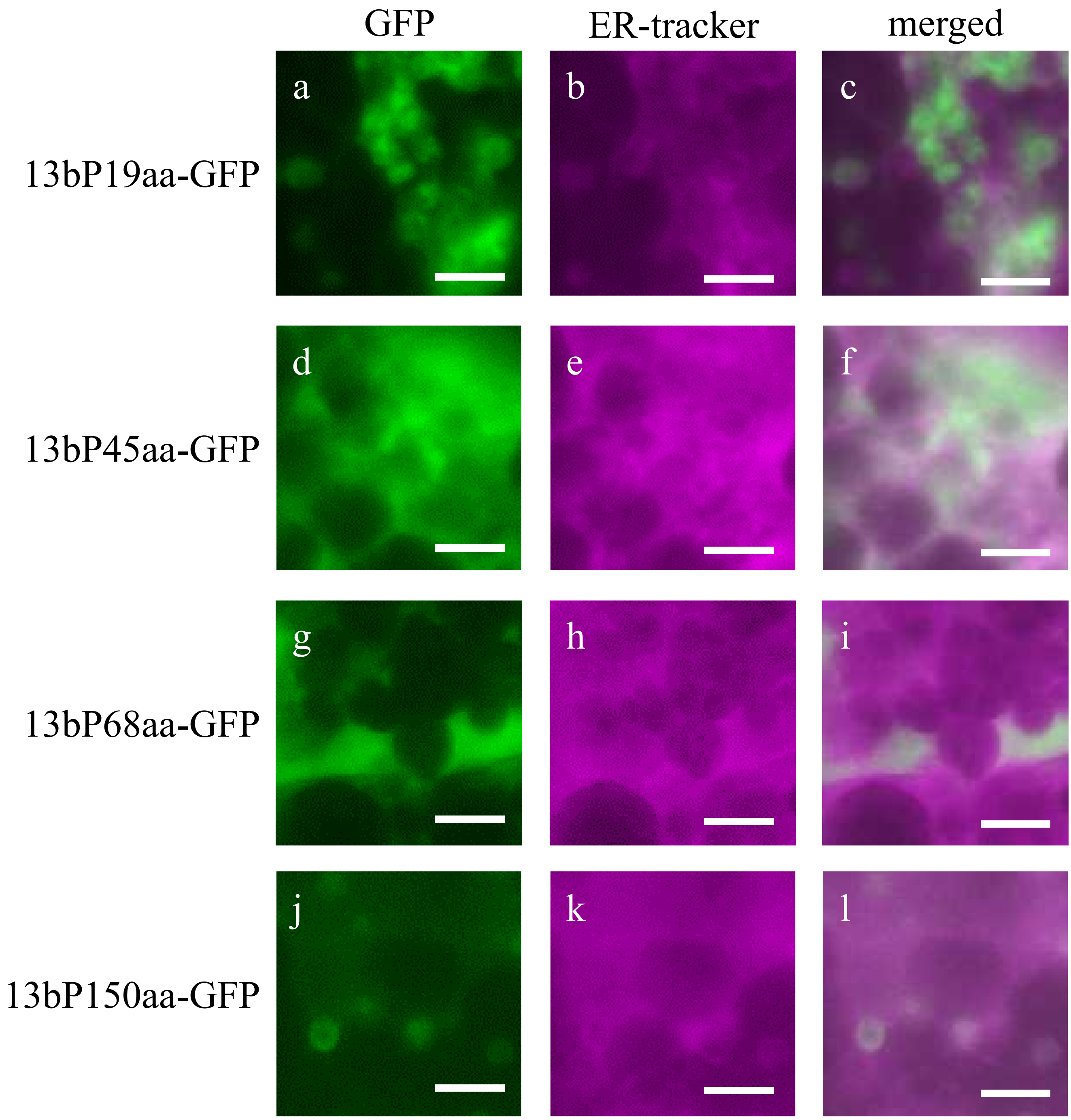

Supplementary Fig. 2. The accumulations of 13b prolamin portion sequence-GFP fusion proteins in 13bP19aa-GFP, 13bP45aa-GFP, and 13bP68aa-GFP were in the ER. We stained frozen thin sections with 10 nM ER-Tracker^{TM} Red for 20 min. The fluorescence images of mature transgenic rice seeds expressing the 19th aa portion sequence-GFP fusion protein (**a**–**c**), the 45th aa portion sequence-GFP fusion protein(**d**–**f**), the 68th aa sequence-GFP fusion protein (**g**–**i**), and the 150th aa sequence (full length)-GFP fusion protein (**j**–**l**) were captured by fluorescence microscopy. Green images: GFP fluorescent signals (**a**, **d**, **g**, **j**). Magenta images: the fluorescence of ER-Tracker^{TM} Red (**b**, **e**, **h**, **k**). The merged images are **c**, **f**, **i**, and **l**. The signals of GFP are surrounded by ER-Tracker. Bars = 5 μm.

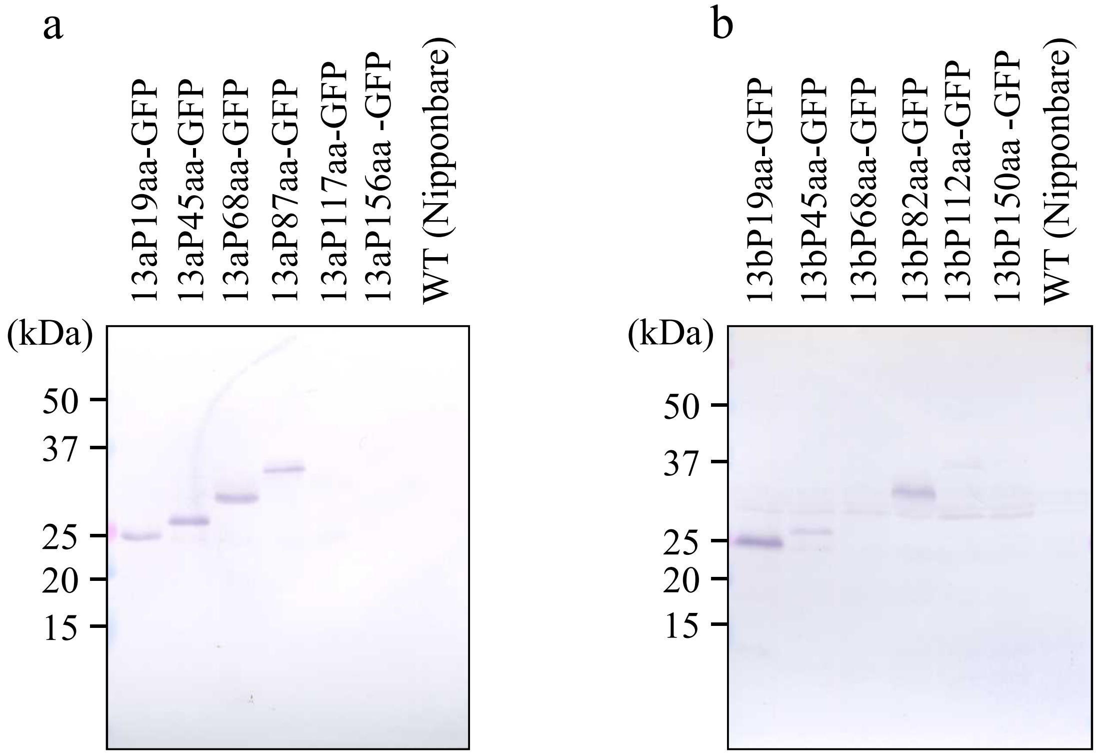

Supplementary Fig. 3. The extraction and detection of each fusion protein using sample buffer without SDS. The detection of 13a prolamin portion sequence-GFP fusion protein (**a**) or 13b portion sequence-GFP fusion protein (**b**) in transgenic rice seeds was confirmed by immunoblotting using anti-GFP antibody.
